# Chloroquine is a potent inhibitor of SARS coronavirus infection and spread

**DOI:** 10.1186/1743-422X-2-69

**Published:** 2005-08-22

**Authors:** Martin J Vincent, Eric Bergeron, Suzanne Benjannet, Bobbie R Erickson, Pierre E Rollin, Thomas G Ksiazek, Nabil G Seidah, Stuart T Nichol

**Affiliations:** 1 Special Pathogens Brach, Division of Viral and Rickettsial Diseases, Centers for Disease Control and Prevention, 1600 Clifton Road, Atlanta, Georgia, 30333, USA; 2 Laboratory of Biochemical Neuroendocrinology, Clinical Research Institute of Montreal, 110 Pine Ave West, Montreal, QCH2W1R7, Canada

**Keywords:** severe acute respiratory syndrome coronavirus, chloroquine, inhibition, therapy

## Abstract

**Background:**

Severe acute respiratory syndrome (SARS) is caused by a newly discovered coronavirus (SARS-CoV). No effective prophylactic or post-exposure therapy is currently available.

**Results:**

We report, however, that chloroquine has strong antiviral effects on SARS-CoV infection of primate cells. These inhibitory effects are observed when the cells are treated with the drug either before or after exposure to the virus, suggesting both prophylactic and therapeutic advantage. In addition to the well-known functions of chloroquine such as elevations of endosomal pH, the drug appears to interfere with terminal glycosylation of the cellular receptor, angiotensin-converting enzyme 2. This may negatively influence the virus-receptor binding and abrogate the infection, with further ramifications by the elevation of vesicular pH, resulting in the inhibition of infection and spread of SARS CoV at clinically admissible concentrations.

**Conclusion:**

Chloroquine is effective in preventing the spread of SARS CoV in cell culture. Favorable inhibition of virus spread was observed when the cells were either treated with chloroquine prior to or after SARS CoV infection. In addition, the indirect immunofluorescence assay described herein represents a simple and rapid method for screening SARS-CoV antiviral compounds.

## Background

Severe acute respiratory syndrome (SARS) is an emerging disease that was first reported in Guangdong Province, China, in late 2002. The disease rapidly spread to at least 30 countries within months of its first appearance, and concerted worldwide efforts led to the identification of the etiological agent as SARS coronavirus (SARS-CoV), a novel member of the family *Coronaviridae *[1]. Complete genome sequencing of SARS-CoV [2,3] confirmed that this pathogen is not closely related to any of the previously established coronavirus groups. Budding of the SARS-CoV occurs in the Golgi apparatus [4] and results in the incorporation of the envelope spike glycoprotein into the virion. The spike glycoprotein is a type I membrane protein that facilitates viral attachment to the cellular receptor and initiation of infection, and angiotensin-converting enzyme-2 (ACE2) has been identified as a functional cellular receptor of SARS-CoV [5]. We have recently shown that the processing of the spike protein was effected by furin-like convertases and that inhibition of this cleavage by a specific inhibitor abrogated cytopathicity and significantly reduced the virus titer of SARS-CoV [6].

Due to the severity of SARS-CoV infection, the potential for rapid spread of the disease, and the absence of proven effective and safe *in vivo *inhibitors of the virus, it is important to identify drugs that can effectively be used to treat or prevent potential SARS-CoV infections. Many novel therapeutic approaches have been evaluated in laboratory studies of SARS-CoV: notable among these approaches are those using siRNA [7], passive antibody transfer [8], DNA vaccination [9], vaccinia or parainfluenza virus expressing the spike protein [10,11], interferons [12,13], and monoclonal antibody to the S1-subunit of the spike glycoprotein that blocks receptor binding [14]. In this report, we describe the identification of chloroquine as an effective pre- and post-infection antiviral agent for SARS-CoV. Chloroquine, a 9-aminoquinoline that was identified in 1934, is a weak base that increases the pH of acidic vesicles. When added extracellularly, the non-protonated portion of chloroquine enters the cell, where it becomes protonated and concentrated in acidic, low-pH organelles, such as endosomes, Golgi vesicles, and lysosomes. Chloroquine can affect virus infection in many ways, and the antiviral effect depends in part on the extent to which the virus utilizes endosomes for entry. Chloroquine has been widely used to treat human diseases, such as malaria, amoebiosis, HIV, and autoimmune diseases, without significant detrimental side effects [15]. Together with data presented here, showing virus inhibition in cell culture by chloroquine doses compatible with patient treatment, these features suggest that further evaluation of chloroquine in animal models of SARS-CoV infection would be warranted as we progress toward finding effective antivirals for prevention or treatment of the disease.

## Results

### Preinfection chloroquine treatment renders Vero E6 cells refractory to SARS-CoV infection

**Figure 1:**
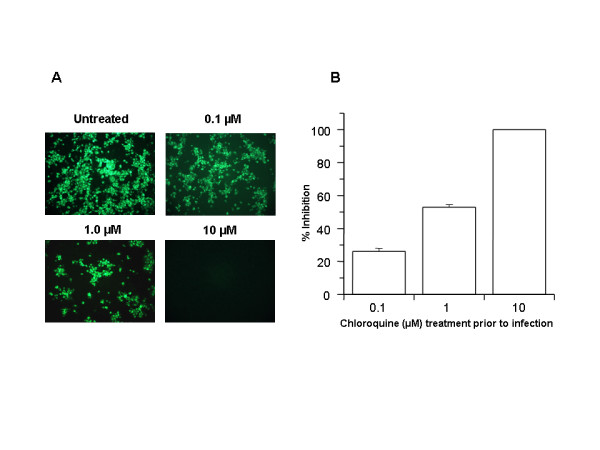
**Prophylactic effect of chloroquine**. Vero E6 cells pre-treated with chloroquine for 20 hrs. Chloroquine-containing media were removed and the cells were washed with phosphate buffered saline before they were infected with SARS-CoV (0.5 multiplicity of infection) for 1 h. in the absence of chloroquine. Virus was then removed and the cells were maintained in Opti-MEM (Invitrogen) for 16–18 h in the absence of chloroquine. SARS-CoV antigens were stained with virus-specific HMAF, followed by FITC-conjugated secondary antibodies. **(A) **The concentration of chloroquine used is indicated on the top of each panel. **(B) **SARS-CoV antigen-positive cells at three random locations were captured by using a digital camera, the number of antigen-positive cells was determined, and the average inhibition was calculated. Percent inhibition was obtained by considering the untreated control as 0% inhibition. The vertical bars represent the range of SEM.

In order to investigate if chloroquine might prevent SARS-CoV infection, permissive Vero E6 cells [1] were pretreated with various concentrations of chloroquine (0.1–10 μM) for 20–24 h prior to virus infection. Cells were then infected with SARS-CoV, and virus antigens were visualized by indirect immunofluorescence as described in Materials and Methods. Microscopic examination (Fig. 1A) of the control cells (untreated, infected) revealed extensive SARS-CoV-specific immunostaining of the monolayer. A dose-dependant decrease in virus antigen-positive cells was observed starting at 0.1 μM chloroquine, and concentrations of 10 μM completely abolished SARS-CoV infection. For quantitative purposes, we counted the number of cells stained positive from three random locations on a slide. The average number of positively stained control cells was scored as 100% and was compared with the number of positive cells observed under various chloroquine concentrations (Fig. 1B). Pretreatment with 0.1, 1, and 10 μM chloroquine reduced infectivity by 28%, 53%, and 100%, respectively. Reproducible results were obtained from three independent experiments. These data demonstrated that pretreatment of Vero E6 cells with chloroquine rendered these cells refractory to SARS-CoV infection.

### Postinfection chloroquine treatment is effective in preventing the spread of SARS-CoV infection

**Figure 2:**
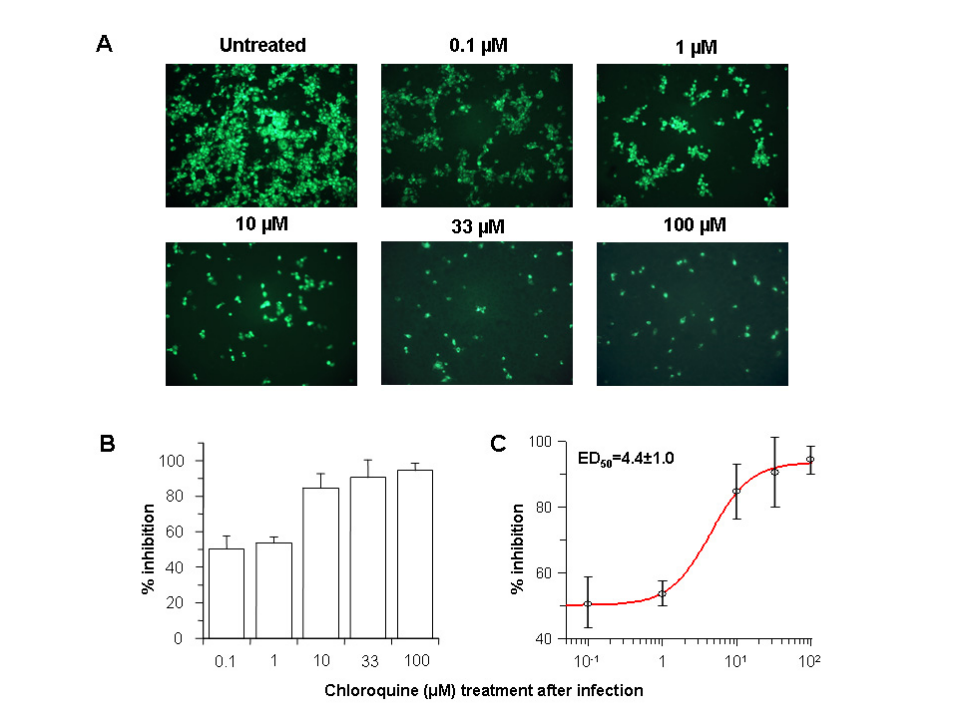
**Post-infection chloroquine treatment reduces SARS-CoV infection and spread**. Vero E6 cells were seeded and infected as described for Fig. 1 except that chloroquine was added only after virus adsorption. Cells were maintained in Opti-MEM (Invitrogen) containing chloroquine for 16–18 h, after which they were processed for immunofluorescence. **(A) **The concentration of chloroquine is indicated on the top. **(B) **Percent inhibition and SEM were calculated as in Fig. 1B. **(C) **The effective dose (ED_50_) was calculated using commercially available software (Grafit, version 4, Erithacus Software).

In order to investigate the antiviral properties of chloroquine on SARS-CoV after the initiation of infection, Vero E6 cells were infected with the virus and fresh medium supplemented with various concentrations of chloroquine was added immediately after virus adsorption. Infected cells were incubated for an additional 16–18 h, after which the presence of virus antigens was analyzed by indirect immunofluorescence analysis. When chloroquine was added after the initiation of infection, there was a dramatic dose-dependant decrease in the number of virus antigen-positive cells (Fig. 2A). As little as 0.1–1 μM chloroquine reduced the infection by 50% and up to 90–94% inhibition was observed with 33–100 μM concentrations (Fig. 2B). At concentrations of chloroquine in excess of 1 μM, only a small number of individual cells were initially infected, and the spread of the infection to adjacent cells was all but eliminated. A half-maximal inhibitory effect was estimated to occur at 4.4 ± 1.0 μM chloroquine (Fig. 2C). These data clearly show that addition of chloroquine can effectively reduce the establishment of infection and spread of SARS-CoV if the drug is added immediately following virus adsorption.

**Figure 3:**
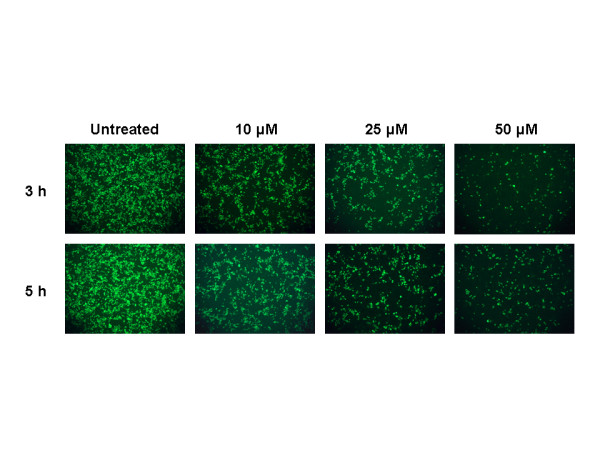
**Timed post-infection treatment with chloroquine**. This experiment is similar to that depicted in Fig. 2 except that cells were infected at 1 multiplicity of infection, and chloroquine (10, 25, and 50 μM) was added 3 or 5 h after infection.

Electron microscopic analysis indicated the appearance of significant amounts of extracellular virus particles 5–6 h after infection [16]. Since we observed antiviral effects by chloroquine immediately after virus adsorption, we further extended the analysis by adding chloroquine 3 and 5 h after virus adsorption and examined for the presence of virus antigens after 20 h. We found that chloroquine was still significantly effective even when added 5 h after infection (Fig. 3); however, to obtain equivalent antiviral effect, a higher concentration of chloroquine was required if the drug was added 3 or 5 h after adsorption.

### Ammonium chloride inhibits SARS-CoV infection of Vero E6 cells

**Figure 4:**
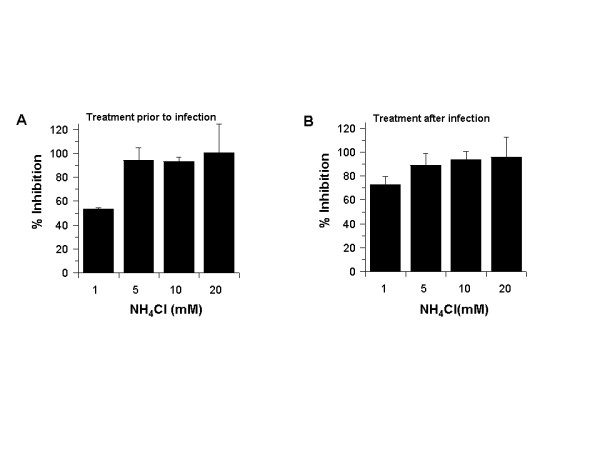
**NH_4_Cl inhibits SARS-CoV during pre or post infection treatment**. NH_4_Cl was added to the cells either before (A) or after (B) infection, similar to what was done for chloroquine in Figs 1 and 2. Antigen-positive cells were counted, and the results were presented as in Fig. 1B.

Since chloroquine inhibited SARS-CoV infection when added before or after infection, we hypothesized that another common lysosomotropic agent, NH_4_Cl, might also function in a similar manner. Ammonium chloride has been widely used in studies addressing endosome-mediated virus entry. Coincidently, NH_4_Cl was recently shown to reduce the transduction of pseudotype viruses decorated with SARS-CoV spike protein [17,18]. In an attempt to examine if NH_4_Cl functions similarly to chloroquine, we performed infection analyses in Vero E6 cells before (Fig. 4A) and after (Fig. 4B) they were treated with various concentrations of NH_4_Cl. In both cases, we observed a 93–99% inhibition with NH_4_Cl at ≥ 5 mM. These data indicated that NH_4_Cl (≥ 5 mM) and chloroquine (≥ 10 μM) are very effective in reducing SARS-CoV infection. These results suggest that effects of chloroquine and NH_4_Cl in controlling SARS CoV infection and spread might be mediated by similar mechanism(s).

### Effect of chloroquine and NH_4_Cl on cell surface expression of ACE2

**Figure 5:**
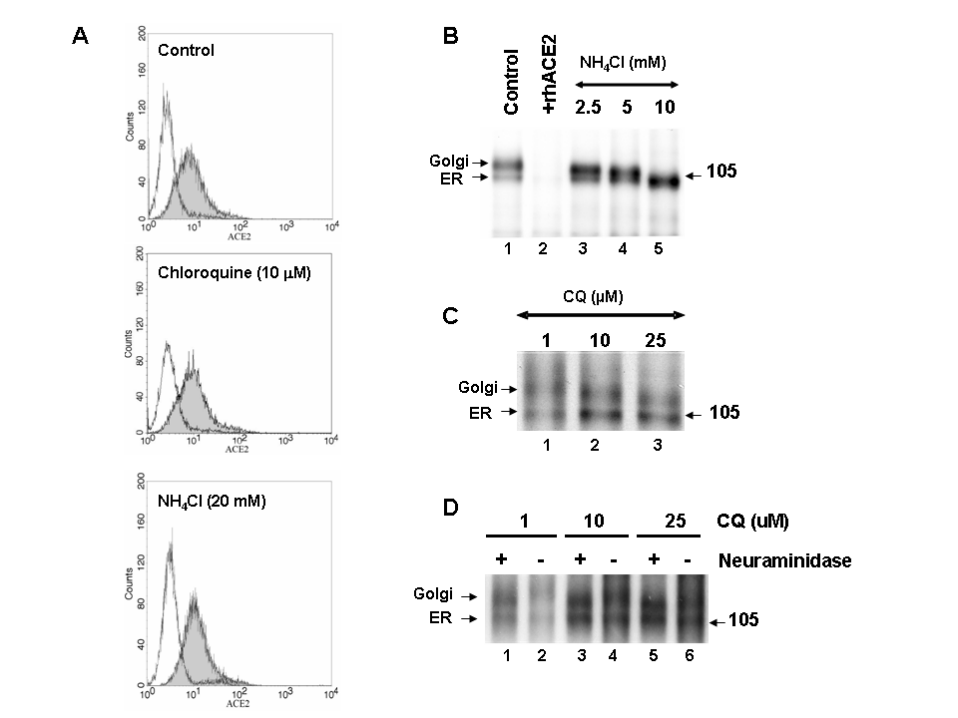
**Effect of lysomotropic agents on the cell-surface expression and biosynthesis of ACE2**. **(A) **Vero E6 cells were cultured for 20 h in the absence (control) or presence of chloroquine (10 μM) or NH_4_Cl (20 mM). Cells were labeled with anti-ACE2 (grey histogram) or with a secondary antibody alone (white histogram). **(B) **Biosynthesis of ACE2 in untreated cells or in cells treated with NH_4_Cl. Vero E6 cells were pulse-labeled for 3 h with ^35^S-Met, and the cell lysates were immunoprecipitated with an ACE2 antibody (lane 1). Preincunbation of the antibody with recombinant human ACE2 (rhACE2) completely abolished the signal (lane 2). The positions of the endoglycosidase H-sensitive ER form and the endoglycosidase H-resistant Golgi form of ACE2 are emphasized. Note that the increasing concentration of NH_4_Cl resulting in the decrease of the Golgi form of ACE2. **(C) **A similar experiment was performed in the presence of the indicated concentrations of chloroquine. Note the loss of terminal glycans with increasing concentrations of chloroquine. **(D) **The terminal glycosidic modification of ACE2 was evaluated by neuraminidase treatment of immunoprecipitated ACE2. Here cells were treated with 1–25 μM concentrations of chloroquine during starvation, pulse, and 3-h chase.

We performed additional experiments to elucidate the mechanism of SARS-CoV inhibition by chloroquine and NH_4_Cl. Since intra-vesicular acidic pH regulates cellular functions, including N-glycosylation trimming, cellular trafficking, and various enzymatic activities, it was of interest to characterize the effect of both drugs on the processing, glycosylation, and cellular sorting of SARS-CoV spike glycoprotein and its receptor, ACE2. Flow cytometry analysis was performed on Vero E6 cells that were either untreated or treated with highly effective anti-SARS-CoV concentrations of chloroquine or NH_4_Cl. The results revealed that neither drug caused a significant change in the levels of cell-surface ACE2, indicating that the observed inhibitory effects on SARS-CoV infection are not due to the lack of available cell-surface ACE2 (Fig. 5A). We next analyzed the molecular forms of endogenous ACE2 in untreated Vero E6 cells and in cells that were pre-incubated for 1 h with various concentrations of either NH_4_Cl (2.5–10 mM) or chloroquine (1 and 10 μM) and labeled with ^35^S-(Met) for 3 h in the presence or absence of the drugs (Fig. 5B and 5C). Under normal conditions, we observed two immunoreactive ACE2 forms, migrating at ~105 and ~113 kDa, respectively (Fig. 5B, lane 1). The ~105-kDa protein is endoglycosidase H sensitive, suggesting that it represents the endoplasmic reticulum (ER) localized form, whereas the ~113-kDa protein is endoglycosidase H resistant and represents the Golgi-modified form of ACE2 [19]. The specificity of the antibody was confirmed by displacing the immunoreactive protein bands with excess cold-soluble human recombinant ACE2 (+ rhACE2; Fig. 5B, lane 2). When we analyzed ACE2 forms in the presence of NH_4_Cl, a clear stepwise increase in the migration of the ~113-kDa protein was observed with increasing concentrations of NH_4_Cl, with a maximal effect observed at 10 mM NH_4_Cl, resulting in only the ER form of ACE2 being visible on the gel (Fig. 5B, compare lanes 3–5). This suggested that the trimming and/or terminal modifications of the N-glycosylated chains of ACE2 were affected by NH_4_Cl treatment. In addition, at 10 mM NH_4_Cl, the ER form of ACE2 migrated with slightly faster mobility, indicating that NH_4_Cl at that concentration might also affect core glycosylation. We also examined the terminal glycosylation status of ACE2 when the cells were treated with chloroquine (Fig. 5C). Similar to NH_4_Cl, a stepwise increase in the electrophoretic mobility of ACE2 was observed with increasing concentrations of chloroquine. At 25 μM chloroquine, the faster electrophoretic mobility of the Golgi-modified form of ACE2 was clearly evident. On the basis of the flow cytometry and immunoprecipitation analyses, it can be inferred that NH_4_Cl and chloroquine both impaired the terminal glycosylation of ACE2, while NH_4_Cl resulted in a more dramatic effect. Although ACE2 is expressed in similar quantities at the cell surface, the variations in its glycosylation status might render the ACE2-SARS-CoV interaction less efficient and inhibit virus entry when the cells are treated with NH_4_Cl and chloroquine.

To confirm that ACE2 undergoes terminal sugar modifications and that the terminal glycosylation is affected by NH_4_Cl or chloroquine treatment, we performed immunopreipitation of ^35^S-labeled ACE2 and subjected the immunoprecipitates to neuraminidase digestion. Proteins were resolved using SDS-PAGE (Fig 5D). It is evident from the slightly faster mobility of the Golgi form of ACE2 after neuraminidase treatment (Fig 5D, compare lanes 1 and 2), that ACE2 undergoes terminal glycosylation; however, the ER form of ACE2 was not affected by neuraminidase. Cells treated with 10 μM chloroquine did not result in a significant shift; whereas 25 μM chloroquine caused the Golgi form of ACE2 to resolve similar to the neuraminidase-treated ACE2 (Fig 5D, compare lanes 5 and 6). These data provide evidence that ACE2 undergoes terminal glycosylation and that chloroquine at anti-SARS-CoV concentrations abrogates the process.

### Effect of chloroquine and NH_4_Cl on the biosynthesis and processing of SARS-CoV spike protein

**Figure 6:**
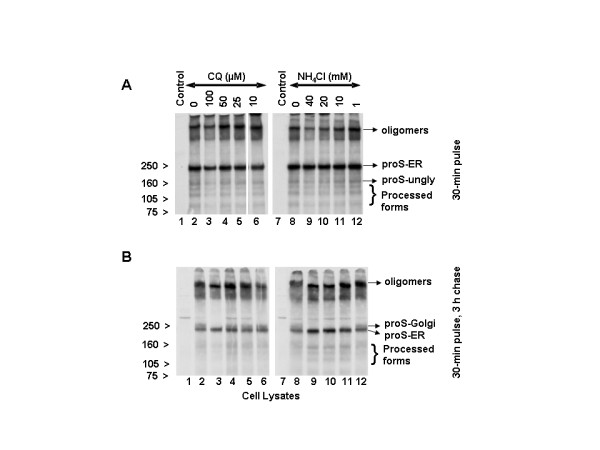
**Effects of NH_4_Cl and chloroquine (CQ) on the biosynthesis, processing, and glycosylation of SARS-CoV spike protein**. Vero E6 cells were infected with SARS-CoV as described in Fig. 2. CQ or NH_4_Cl was added during the periods of starvation (1 h) and labeling (30 min) with ^35^S-Cys and followed by chase for 3 h in the presence of unlabeled medium. Cells were lysed in RIPA buffer and immunoprecipitated with HMAF. Virus proteins were resolved using 3–8% NuPAGE gel (Invitrogen). The cells presented were labeled for 30 min **(A) **and chased for 3 h **(B)**. The migration positions of the various spike molecular forms are indicated at the right side, and those of the molecular standards are shown to the left side. proS-ER and proS-Golgi are the pro-spike of SARS-Co in the ER and Golgi compartments, respectively and proS-ungly is the unglycosylated pro-spike ER.

We next addressed whether the lysosomotropic drugs (NH_4_Cl and chloroquine) affect the biosynthesis, glycosylation, and/or trafficking of the SARS-CoV spike glycoprotein. For this purpose, Vero E6 cells were infected with SARS-CoV for 18 h. Chloroquine or ammonium chloride was added to these cells during while they were being starved (1 h), labeled (30 min) or chased (3 h). The cell lysates were analyzed by immunoprecipitation with the SARS-specific polyclonal antibody (HMAF). The 30-min pulse results indicated that pro-spike (proS) was synthesized as a ~190-kDa precursor (proS-ER) and processed into ~125-, ~105-, and ~80-kDa proteins (Fig. 6A, lane 2), a result identical to that in our previous analysis [6]. Except for the 100 μM chloroquine (Fig. 6A, lane 3), there was no significant difference in the biosynthesis or processing of the virus spike protein in untreated or chloroquine-treated cells (Fig. 6A, lanes 4–6). It should be noted that chloroquine at 100 μM resulted in an overall decrease in biosynthesis and in the levels of processed virus glycoprotein. In view of the lack of reduction in the biosynthesis and processing of the spike glycoprotein in the presence of chloroquine concentrations (10 and 50 μM) that caused large reductions in SARS-CoV replication and spread, we conclude that the antiviral effect is probably not due to alteration of virus glycoprotein biosynthesis and processing. Similar analyses were performed with NH_4_Cl, and the data suggested that the biosynthesis and processing of the spike protein were also not negatively affected by NH_4_Cl (Fig. 6A, lanes 7–12). Consistent with our previous analysis [6], we observed the presence of a larger protein, which is referred to here as oligomers. Recently, Song et al. [20] provided evidence that these are homotrimers of the SARS-CoV spike protein and were incorporated into the virions. Interestingly, the levels of the homotrimers in cells treated with 100 μM chloroquine and 40 and 20 mM NH_4_Cl (Fig. 6A, lanes 3, 9, and 10) were slightly lower than in control cells or cells treated with lower drug concentrations.

The data obtained from a 30-min pulse followed by a 3-h chase (Fig. 6B, lanes 2 and 8) confirmed our earlier observation that the SARS-CoV spike protein precursor (proS-ER) acquires Golgi-specific modifications (proS-Golgi) resulting in a ~210-kDa protein [6]. Chloroquine at 10, 25, and 50 μM had no substantial negative impact on the appearance of the Golgi form (Fig. 6B, compare lane 2 to lanes 4–6). Only at 100 μM chloroquine was a reduction in the level of the Golgi-modified pro-spike observed (lane 3). On the other hand, NH_4_Cl abrogated the appearance of Golgi-modified forms at ≥10 mM (compare lane 8 with 9–11) and had a milder effect at 1 mM (lane 12). These data clearly demonstrate that the biosynthesis and proteolytic processing of SARS-CoV spike protein are not affected at chloroquine (25 and 50 μM) and NH_4_Cl (1 mM) doses that cause virus inhibitory effects. In addition, with 40, 20, and 10 mM NH_4_Cl, there was an increased accumulation of proS-ER with a concomitant decrease in the amount of oligomers (Fig. 6B, lanes 9–11). When we examined the homotrimers, we found that chloroquine at 100 μM and NH_4_Cl at 40 and 20 mM resulted in slightly faster mobility of the trimers (Fig. 6B, lanes 3, 9, and 10), but lower drug doses, which did exhibit significant antiviral effects, did not result in appreciable differences. These data suggest that the newly synthesized intracellular spike protein may not be a major target for chloroquine and NH_4_Cl antiviral action. The faster mobility of the trimer at certain higher concentration of the drugs might be due the effect of these drugs on the terminal glycosylation of the trimers.

## Discussion

We have identified chloroquine as an effective antiviral agent for SARS-CoV in cell culture conditions, as evidenced by its inhibitory effect when the drug was added prior to infection or after the initiation and establishment of infection. The fact that chloroquine exerts an antiviral effect during pre- and post-infection conditions suggest that it is likely to have both prophylactic and therapeutic advantages. Recently, Keyaerts et al. [21] reported the antiviral properties of chloroquine and identified that the drug affects SARS-CoV replication in cell culture, as evidenced by quantitative RT-PCR. Taken together with the findings of Keyaerts et al. [21], our analysis provides further evidence that chloroquine is effective against SARS-CoV Frankfurt and Urbani strains. We have provided evidence that chloroquine is effective in preventing SARS-CoV infection in cell culture if the drug is added to the cells 24 h prior to infection. In addition, chloroquine was significantly effective even when the drug was added 3–5 h after infection, suggesting an antiviral effect even after the establishment of infection. Since similar results were obtained by NH_4_Cl treatment of Vero E6 cells, the underlying mechanism(s) of action of these drugs might be similar.

Apart from the probable role of chloroquine on SARS-CoV replication, the mechanisms of action of chloroquine on SARS-CoV are not fully understood. Previous studies have suggested the elevation of pH as a mechanism by which chloroquine reduces the transduction of SARS-CoV pseudotype viruses [17,18]. We examined the effect of chloroquine and NH_4_Cl on the SARS-CoV spike proteins and on its receptor, ACE2. Immunoprecipitation results of ACE2 clearly demonstrated that effective anti-SARS-CoV concentrations of chloroquine and NH_4_Cl also impaired the terminal glycosylation of ACE2. However, the flow cytometry data demonstrated that there are no significant differences in the cell surface expression of ACE2 in cells treated with chloroquine or NH_4_Cl. On the basis of these results, it is reasonable to suggest that the pre-treatment with NH_4_Cl or chloroquine has possibly resulted in the surface expression of the under-glycosylated ACE2. In the case of chloroquine treatment prior to infection, the impairment of terminal glycosylation of ACE2 may result in reduced binding affinities between ACE2 and SARS-CoV spike protein and negatively influence the initiation of SARS-CoV infection. Since the biosynthesis, processing, Golgi modification, and oligomerization of the newly synthesized spike protein were not appreciably affected by anti-SARS-CoV concentrations of either chloroquine or NH_4_Cl, we conclude that these events occur in the cell independent of the presence of the drugs. The potential contribution of these drugs in the elevation of endosomal pH and its impact on subsequent virus entry or exit could not be ruled out. A decrease in SARS-CoV pseudotype transduction in the presence of NH_4_Cl was observed and was attributed to the effect on intracellular pH [17,18]. When chloroquine or NH_4_Cl are added after infection, these agents can rapidly raise the pH and subvert on-going fusion events between virus and endosomes, thus inhibiting the infection.

In addition, the mechanism of action of NH_4_Cl and chloroquine might depend on when they were added to the cells. When added after the initiation of infection, these drugs might affect the endosome-mediated fusion, subsequent virus replication, or assembly and release. Previous studies of chloroquine have demonstrated that it has multiple effects on mammalian cells in addition to the elevation of endosomal pH, including the prevention of terminal glycosyaltion of immunoglobulins [22]. When added to virus-infected cells, chloroquine inhibited later stages in vesicular stomatitis virus maturation by inhibiting the glycoprotein expression at the cell surface [23], and it inhibited the production of infectious HIV-1 particles by interfering with terminal glycosylation of the glycoprotein [24,25]. On the basis of these properties, we suggest that the cell surface expression of under-glycosylated ACE2 and its poor affinity to SARS-CoV spike protein may be the primary mechanism by which infection is prevented by drug pretreatment of cells prior to infection. On the other hand, rapid elevation of endosomal pH and abrogation of virus-endosome fusion may be the primary mechanism by which virus infection is prevented under post-treatment conditions. More detailed SARS CoV spike-ACE2 binding assays in the presence or absence of chloroquine will be performed to confirm our findings. Our studies indicate that the impact of NH_4_Cl and chloroquine on the ACE2 and spike protein profiles are significantly different. NH_4_Cl exhibits a more pronounced effect than does chloroquine on terminal glycosylation, highlighting the novel intricate differences between chloroquine and ammonium chloride in affecting the protein transport or glycosylation of SARS-CoV spike protein and its receptor, ACE2, despite their well-established similar effects of endosomal pH elevation.

The infectivity of coronaviruses other than SARS-CoV are also affected by chloroquine, as exemplified by the human CoV-229E [15]. The inhibitory effects observed on SARS-CoV infectivity and cell spread occurred in the presence of 1–10 μM chloroquine, which are plasma concentrations achievable during the prophylaxis and treatment of malaria (varying from 1.6–12.5 μM) [26] and hence are well tolerated by patients. It recently was speculated that chloroquine might be effective against SARS and the authors suggested that this compound might block the production of TNFα, IL6, or IFNγ [15]. Our data provide evidence for the possibility of using the well-established drug chloroquine in the clinical management of SARS.

## Conclusion

Chloroquine, a relatively safe, effective and cheap drug used for treating many human diseases including malaria, amoebiosis and human immunodeficiency virus is effective in inhibiting the infection and spread of SARS CoV in cell culture. The fact that the drug has significant inhibitory antiviral effect when the susceptible cells were treated either prior to or after infection suggests a possible prophylactic and therapeutic use.

## Methods

### SARS-CoV infection, immunofluorescence, and immunoprecipitation analyses

Vero E6 cells (an African green monkey kidney cell line) were infected with SARS-CoV (Urbani strain) at a multiplicity of infection of 0.5 for 1 h. The cells were washed with PBS and then incubated in OPTI-MEM (Invitrogen) medium with or without various concentrations of either chloroquine or NH_4_Cl (both from Sigma). Immunofluorescence staining was performed with SARS-CoV-specific hyperimmune mouse ascitic fluid (HMAF) [8] followed by anti-mouse fluorescein-coupled antibody.

Eighteen hours after infection, the virus-containing supernatants were removed, and the cells were pulsed with ^35^S-(Cys) for 30 min and chased for 3 h before lysis in RIPA buffer. Clarified cell lysates and media were incubated with HMAF, and immunoprecipitated proteins were separated by 3–8% NuPAGE gel (Invitrogen); proteins were visualized by autoradiography. In some experiments, cells were chased for 3 h with isotope-free medium. Clarified cell supernatants were also immunoprecipitated with SARS-CoV-specific HMAF.

### ACE2 flow cytometry analysis and biosynthesis

Vero E6 cells were seeded in Dulbecco's modified Eagle medium (Invitrogen) supplemented with 10% fetal bovine serum. The next day, the cells were incubated in Opti-MEM (Invitrogen) in the presence or absence of 10 μM chloroquine or 20 mM NH_4_Cl. To analyze the levels of ACE2 at the cell surface, cells were incubated on ice with 10 μg/mL affinity-purified goat anti-ACE2 antibody (R&D Systems) and then incubated with FITC-labeled swine anti-goat IgG antibody (Caltag Laboratories). Labeled cells were analyzed by flow cytometry with a FACSCalibur flow cytometer (BD Biosciences). For ACE2 biosynthesis studies, Vero E6 cells were pulsed with 250 μCi ^35^S-(Met) (Perkin Elmer) for 3 h with the indicated concentrations of chloroquine or NH_4_Cl and then lysed in RIPA buffer. Clarified lysates were immunoprecipitated with an affinity-purified goat anti-ACE2 antibody (R&D systems), and the immunoprecipitated proteins were separated by SDS-polyacrylamide gel electrophoresis.

## Competing interests

The author(s) declare that they have no competing interests.

## Authors' contributions

MV did all the experiments pertaining to SARS CoV infection and coordinated the drafting of the manuscript. EB and SB performed experiments on ACE2 biosynthesis and FACS analysis. BE performed data acquisition from the immunofluorescence experiments. PR and TK provided critical reagents and revised the manuscript critically. NS and SN along with MV and EB participated in the planning of the experiments, review and interpretation of data and critical review of the manuscript. All authors read and approved the content of the manuscript.

## References

[B1] Ksiazek TG, Erdman D, Goldsmith CS, Zaki SR, Peret T, Emery S, Tong S, Urbani C, Comer JA, Lim W, Rollin PE, Dowell SF, Ling AE, Humphrey CD, Shieh WJ, Guarner J, Paddock CD, Rota PB, Fields B, DeRisi J, Yang JY, Cox N, Hughes J, LeDuc JW, Bellini WJ, Anderson LJ, SARS Working Group (2003). A novel coronavirus associated with severe acute respiratory syndrome. N Engl J Med.

[B2] Marra MA, Jones SJ, Astell CR, Holt RA, Brooks-Wilson A, Butterfield YS, Khattra J, Asano JK, Barber SA, Chan SY, Cloutier A, Coughlin SM, Freeman D, Girn N, Griffith OL, Leach SR, Mayo, McDonald H, Montgomery SB, Pandoh PK, Petrescu AS, Robertson AG, Schein JE, Siddiqui A, Smailus DE, Stott JM, Yang GS, Plummer F, Andonov A, Artsob H, Bastien N, Bernard K, Booth TF, Bowness D, Czub M, Drebot M, Fernando L, Flick R, Garbutt M, Gray M, Grolla A, Jones S, Feldmann H, Meyers A, Kabani A, Li Y, Normand S, Stroher U, Tipples GA, Tyler S, Vogrig R, Ward D, Watson B, Brunham RC, Krajden M, Petric M, Skowronski DM, Upton C, Roper RL (2003). The Genome sequence of the SARS-associated coronavirus. Science.

[B3] Rota PA, Oberste MS, Monroe SS, Nix WA, Campagnoli R, Icenogle JP, Penaranda S, Bankamp B, Maher K, Chen MH, Tong S, Tamin A, Lowe L, Frace M, DeRisi JL, Chen Q, Wang D, Erdman DD, Peret TC, Burns C, Ksiazek TG, Rollin PE, Sanchez A, Liffick S, Holloway B, Limor J, McCaustland K, Olsen Rasmussen M, Fouchier R, Gunther S, Osterhaus AS, Drosten C, Pallansch MA, Anderson LJ, Bellini WJ (2003). Characterization of a novel coronavirus associated with severe acute respiratory syndrome. Science.

[B4] Ng ML, Tan SH, See EE, Ooi EE, Ling AE (2003). Proliferative growth of SARS coronavirus in Vero E6 cells. J Gen Virol.

[B5] Li M, Moore WJ, Vasilieva N, Sui J, Wong SK, Berne MA, Somasundaran M, Sullivan JL, Luzuriaga K, Greenough TC, Choe H, Farzan M (2003). Angiotensin-converting enzyme 2 is a functional receptor for the SARS coronavirus. Nature.

[B6] Bergeron E, Vincent MJ, Wickham L, Hamelin J, Basak A, Nichol ST, Chrétien M, NG Seidah (2005). Implication of proprotein convertases in the processing and spread of severe acute respiratory syndrome coronavirus. Biochem Biophys Res Comm.

[B7] Zhang Y, Li T, Fu L, Yu C, Li Y, Xu X, Wang Y, Ning H, Zhang S, Chen W, Babiuk LA, Chang Z (2004). Silencing SARS-CoV spike protein expression in cultured cells by RNA interference. FEBS Lett.

[B8] Subbarao K, McAuliffe J, Vogel L, Fahle G, Fischer S, Tatti K, Packard M, Shieh WJ, Zaki S, Murphy B (2004). Prior infection and passive transfer of neutralizing antibody prevent replication of severe acute respiratory syndrome coronavirus in the respiratory tract of mice. J Virol.

[B9] Yang ZY, Kong WP, Huang Y, Roberts A, Murphy BR, Subbarao K, Nabel GJ (2004). A DNA vaccine induces SARS coronavirus neutralization and protective immunity in mice. Nature.

[B10] Bisht H, Roberts A, Vogel L, Bukreyev A, Collins PL, Murphy BR, Subbarao K, Moss B (2004). Severe acute respiratory syndrome coronavirus spike protein expressed by attenuated vaccinia virus protectively immunizes mice. Proc Natl Acad Sci USA.

[B11] Bukreyev A, Lamirande EW, Buchholz UJ, Vogel LN, Elkins WR, St. Claire M, Murphy BR, Subbarao K, Collins PL (2004). Mucosal immunization of African green monkeys (Cercopithecus aethiops) with an attenuated parainfluenza virus expressing the SARS coronavirus spike protein for the prevention of SARS. Lancet.

[B12] Sainz B, Mossel EC, Peters CJ, Garry RF (2004). Interferon-beta and interferon-gamma synergistically inhibit the replication of severe acute respiratory syndrome-associated coronavirus (SARS-CoV). Virology.

[B13] Stroher U, DiCaro A, Li Y, Strong JE, Aoki F, Plummer F, Jones SM, Feldmann H (2004). Severe acute respiratory syndrome-related coronavirus is inhibited by interferon- alpha. J Infect Dis.

[B14] Sui J, Li W, Murakami A, Tamin A, Matthews LJ, Wong SK, Moore MJ, Tallarico AS, Olurinde M, Choe H, Anderson LJ, Bellini WJ, Farzan M, Marasco WA (2004). Potent neutralization of severe acute respiratory syndrome (SARS) coronavirus by a human mAb to S1 protein that blocks receptor association. Proc Natl Acad Sci USA.

[B15] Savarino A, Boelaert JR, Cassone A, Majori G, Cauda R (2003). Effects of chloroquine on viral infections: an old drug against today's diseases?. Lancet Infect Dis.

[B16] Ng ML, Tan SH, See EE, Ooi EE, Ling AE (2003). Early events of SARS coronavirus infection in vero cells. J Med Virol.

[B17] Simmons G, Reeves JD, Rennekamp AJ, Amberg SM, Piefer AJ, Bates P (2004). Characterization of severe acute respiratory syndrome-associated coronavirus (SARS-CoV) spike glycoprotein-mediated viral entry. Proc Natl Acad Sci USA.

[B18] Yang ZY, Huang Y, Ganesh L, Leung K, Kong WP, Schwartz O, Subbarao K, Nabel GJ (2004). pH-dependent entry of severe acute respiratory syndrome coronavirus is mediated by the spike glycoprotein and enhanced by dendritic cell transfer through DC-SIGN. J Virol.

[B19] Tipnis SR, Hooper NM, Hyde R, Karran E, Christie G, Turner AJ (2000). A human homolog of angiotensin-converting enzyme. Cloning and functional expression as a captopril-insensitive carboxypeptidase. J Biol Chem.

[B20] Song HC, Seo MY, Stadler K, Yoo BJ, Choo QL, Coates SR, Uematsu Y, Harada T, Greer CE, Polo JM, Pileri P, Eickmann M, Rappuoli R, Abrignani S, Houghton M, Han JH (2004). Synthesis and characterization of a native, oligomeric form of recombinant severe acute respiratory syndrome coronavirus spike glycoprotein. J Virol.

[B21] Keyaerts E, Vijgen L, Maes P, Neyts J, Ranst MV (2004). In vitro inhibition of severe acute respiratory syndrome coronavirus by chloroquine. Biochem Biophys Res Commun.

[B22] Thorens B, Vassalli P (1986). Chloroquine and ammonium chloride prevent terminal glycosylation of immunoglobulins in plasma cells without affecting secretion. Nature.

[B23] Dille BJ, Johnson TC (1982). Inhibition of vesicular stomatitis virus glycoprotein expression by chloroquine. J Gen Virol.

[B24] Tsai WP, Nara PL, Kung HF, Oroszlan S (1990). Inhibition of human immunodeficiency virus infectivity by chloroquine. AIDS Res Hum Retroviruses.

[B25] Savarino A, Lucia MB, Rastrelli E, Rutella S, Golotta C, Morra E, Tamburrini E, Perno CF, Boelaert JR, Sperber K, Cauda RC (2004). Anti-HIV effects of chloroquine: inhibition of viral particle glycosylation and synergism with protease inhibitors. J Acquir Immune Defic Syndr.

[B26] Ducharme J, Farinotti R (1996). Clinical pharmacokinetics and metabolism of chloroquine. Focus on recent advancements. Clin Pharmacokinet.

